# The role of antioxidants in restoring MAPK 14 and a DNA damage marker level following autophagy suppression

**DOI:** 10.1098/rsob.200253

**Published:** 2020-12-09

**Authors:** Abdalla Elbialy

**Affiliations:** Faculty of Veterinary Medicine, Damanhour University, Damanhour 22511, Egypt

**Keywords:** autophagy, oxidative stress, DNA damage, p38, NH4Cl

## Abstract

Autophagy is a lysosomal degradation mechanism for elimination and recycling of damaged intracellular organelles and proteins. Recent studies have shown that autophagy could help reduce oxidative stress by removing oxidized proteins and damaged mitochondria. Autophagy deficiency is associated with the disruption of many intracellular biological processes. Using bioinformatics tools and fibroblast immunostaining technology, I tried to investigate whether oxidative stress is involved in mediating the effect of autophagy suppression on certain cell biological processes and signalling pathways. Many pharmaceutical components have different modes of action to suppress autophagy. In this study, I performed analysis on autophagy suppression induced by neutralizing lysosomal pH (NH_4_Cl and bafilomycin A1). Bioinformatics analysis of GEO data, GSE60570 accession number, revealed that p38 signalling induction and DNA damage response are among the main disrupted signalling pathways in bafilomycin A1-treated RPE-1 cells. Likewise, fibroblast immunostaining showed that autophagy deficiency established by ammonium chloride (NH_4_Cl) has significantly increased P38 signalling, DNA damage marker (H2A.X), and oxidative stress marker (dityrosine). I therefore investigated the role of oxidative stress and whether antioxidants treatment could reverse autophagy suppression effects on p38 signalling and DNA damage response. Importantly, antioxidant treatment clearly restored P38 signalling and H2A.X levels in autophagy-suppressed fibroblast cells. Indicating that oxidative stress might be associated with the harmful effect of autophagy suppression.

## 1. Introduction

Autophagy is a conservative metabolic process for eliminating and recycling dysfunctional and unnecessary components such as damaged cellular organelles and proteins by delivering cytoplasmic damaged cargo for lysosomal degradation. It is a fundamental biological process for the maintenance of intracellular protein homeostasis and cellular integrity in virtually all cells [1–3]. Reduced autophagy has been associated with ageing, and with multiple age-related diseases such as cancer, metabolic and neurodegenerative diseases [3–5].

Meanwhile, oxidative stress, the imbalance between free radicals and antioxidants, and the resulting cellular damage have been widely believed to play an important role in ageing and various age-related diseases [6,7].

Recent reports suggest the presence of complex crosstalk between autophagy and oxidative stress. Autophagy is triggered under stress conditions such as starvation, ischaemia, pathogen infection and oxidative stress [8,9]. Reactive oxygen species (ROS) are highly reactive small molecules capable of oxidizing proteins, lipids and DNA, and their accumulation induces oxidative stress. It is now widely agreed that ROS induce autophagy [10] and that autophagy in turn reduces oxidative damage [11]. Autophagy activation under stress conditions or in response to ROS is therefore primarily a survival mechanism [9].

Moreover, mitophagy, an autophagy pathway that specifically clears damaged and dysfunctional mitochondria, could act as an essential antioxidant pathway [12] by reducing ROS production. The antioxidant role of autophagy has been observed in glomerular capillaries and muscle stem cells [13–15], probably by mitophagy and subsequent reduction of ROS level.

Reduced autophagy is associated with the disruption of many cellular biological processes and the subsequent induction of ageing and multiple age-related diseases [3].

Since autophagy has a fundamental antioxidant role, I tried to investigate whether oxidative stress is involved in mediating the effect of autophagy suppression on cells. This can be done by first identifying the major disrupted biological processes of autophagy-suppressed cells and then examining whether antioxidant therapy could reverse these effects.

Autophagy modulators are numerous and have different mechanisms of action. In this study, I performed analysis on autophagy suppression induced by neutralizing lysosomal pH (NH_4_Cl and bafilomycin A1).

## 2. Results

### 2.1. Transcriptomic analysis of bafilomycin A1-treated RPE-1 cells reveals enrichments of P38 MAPK pathway, DNA damage response and oxidative stress response

Autophagy inhibition may be achieved by compounds that block the fusion of autophagosomes with lysosomes such as bafilomycin (V-ATPase inhibitor) or lysosomal pH-neutralizing compounds such as NH_4_Cl [16]. Autophagy inhibition by lysosomotropic compounds may increase the number of autophagosomes and therefore the LC3 protein or autophagosome quantification does not represent basal autophagy levels in this case [16].

In order to identify perturbed biological processes of autophagy-suppressed cells following treatment with bafilomycin A1, I performed bioinformatics analysis of the RNA seq data available under the Gene Expression Omnibus (GEO) accession number GSE60570 [17]. In their experiment, RPE-1 cells were treated with Bafilomycin A1 or reversine or MG132 to examine autophagosome clearance in aneuploidy.

I obtained RNA seq count data of bafilomycin A1-treated RPE-1 cells for 6 h relative to the control cells and used various bioinformatics tools to detect the major disrupted signalling. First, using the galaxy server, I detected fold change of gene expression and DEGs (differentially expressed genes) using the edgeR Bioconductor package (adj *p*-value < 0.01). The enrichment analysis was then conducted.

**Figure 1.**
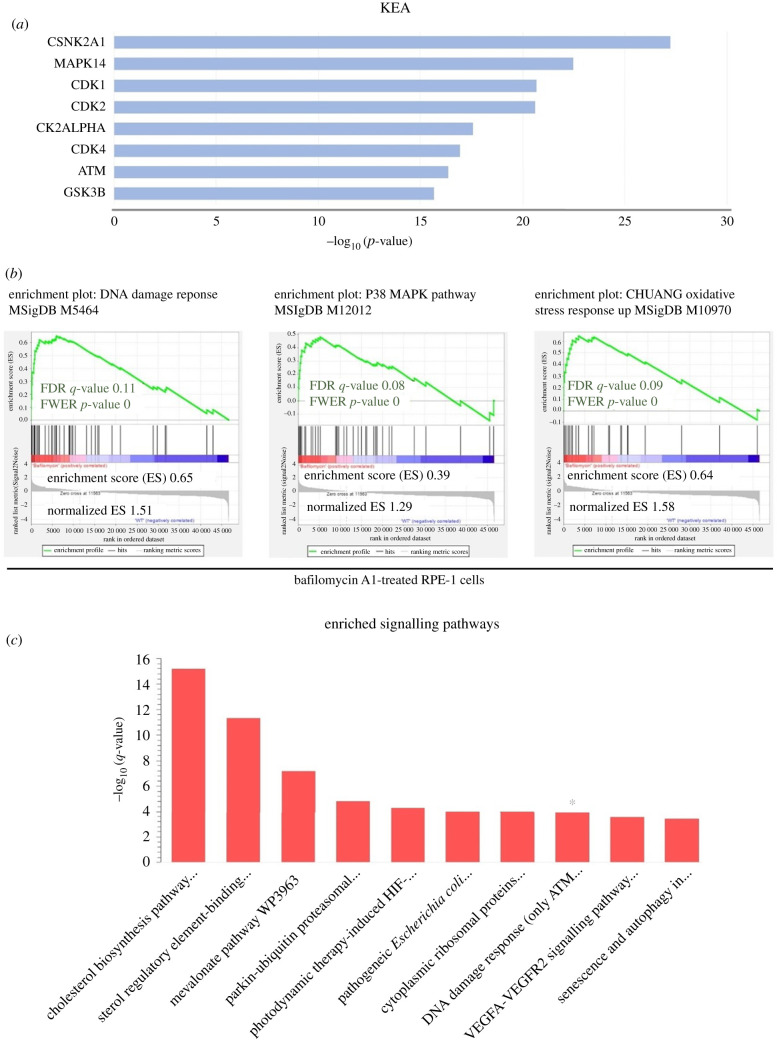
Bafilomycin A1-treatment induces p38 pathway, DNA damage response and oxidative stress response. (*a*) Histogram of KEA results showing the main protein kinases driving the expression of DEGs in bafilomycin A1-treated RPE-1 cells. *y*-axis, the statistical significance of the enrichment; *x*-axis, enriched protein kinases. (*b*) Illustration of statistically significant GSEA results of DNA damage response, P38 pathways and oxidative stress response in bafilomycin A1-treated RPE-1 cells. Significant *p*-values < 0.05 and FDR *q*-values < 0.25 are written in red. The reported *p*-value of 0.0 indicates that the actual *p*-value of less than 0.01 (*n* = 3). (*c*) Histogram for the main enriched signalling pathways of the bafilomycin A1-treated RPE-1 cells from the WikiPathways database. *y*-axis, the statistical significance of the enrichment; *x*-axis, pathway categories. The DNA damage response pathway is denoted by asterisks (*n* = 3).

Kinase enrichment analysis (KEA) analysis showed that the major protein kinases driving the expression of DEGs in bafilomycin A1-treated RPE-1 are involved in the cell cycle (CDK1, CDK2, CDK4, CK2ALPHA, CSNK2A1), DNA damage (ATM) and P38 signalling (MAPK14) (figure 1*a*).

To confirm these results, gene set enrichment analysis (GSEA) analysis was used to test for enrichment against DNA damage response and P38 MAPK pathway datasets. Expectedly, the GSEA analysis revealed a significant upregulation of the DNA damage response and P38 MAPK pathway datasets (*p*-values < 0.05, FDR *q*-values < 0.25) (figure 1*b*). Importantly, the GSEA analysis revealed a significant upregulation of oxidative stress response as well. As can be seen in figure 1*b*, the enrichment score was positive, indicating upregulation of the datasets used.

Additionally, the pathway enrichment analysis of bafilomycin A1-treated RPE-1 cells showed enrichment of the DNA damage response (figure 1*c*). Notably, according to our analysis, other biological themes have been enriched following treatment with bafilomycin A1.

### 2.2. Autophagy deficiency elevates p38 MAPK, DNA damage and oxidative stress markers

In figure 1, transcriptomic analysis of bafilomycin A1-treated RPE-1 cells reveals enrichments of P38 MAPK pathway, DNA damage response and oxidative stress response. To further investigate this point in the laboratory, I used immunocytochemistry to measure dityrosine (oxidative stress marker), phospho-H2A.X protein (DNA damage marker) and p38 signalling levels in NH_4_Cl exposed fibroblast cells.

Since autophagy has a fundamental antioxidant role previously seen in glomerular capillaries and muscle stem cells [13–15], I investigated oxidative stress following autophagy inhibition.

**Figure 2.**
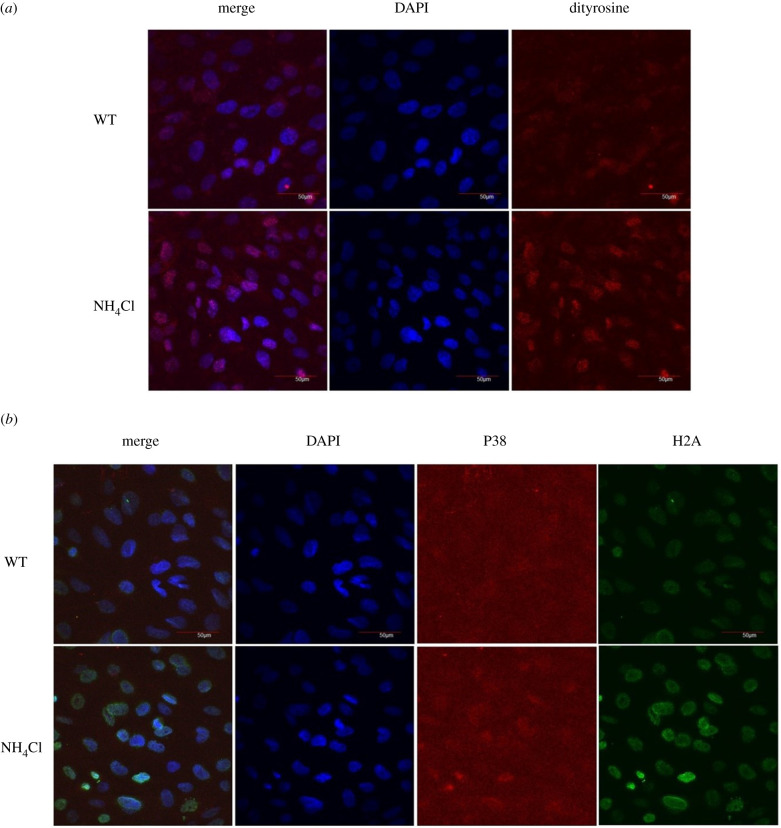
The inhibition of autophagy in fibroblasts induce oxidative stress, p38 signalling and DNA damage. (*a*) Representative dityrosine immunostaining in control and NH_4_Cl-treated groups. (*b*) Representative phospho-H2A.X and P-p38 immunostaining in control and NH_4_Cl-treated groups.

Inhibition of autophagy by neutralizing lysosomal pH following NH_4_Cl treatment of fish fibroblasts substantially enhanced dityrosine staining (oxidative stress marker) (figure 2*a*). Likewise, GSEA analysis of bafilomycin A1-treated RPE-1 cells revealed a significant enrichment of the oxidative stress response (*p*-values < 0.05, FDR *q*-values < 0.25) (figure 1*b*).

Importantly, similar to bafilomycin treatment, Nh4cl treatment progressively increased phospho-H2A.X and P-p38 antibody staining (figure 2*b*).

### 2.3. Kinase enrichment analysis of hydrogen peroxide (H_2_O_2_)-treated Hela cells shows that oxidative stress is involved in p38 signal elevation and DNA damage

Since autophagy suppression increased P38 MAPK pathway, DNA damage response and oxidative stress response (figures 1 and 2), I investigated whether oxidative stress is involved in elevation of p38 signalling and DNA damage response.

I performed RNA seq data analysis of hydrogen peroxide (H_2_O_2_)-treated Hela cells. The raw RNA seq data available on the NCBI SRA accession number SRP140470 were used to retrieve DEGs between exposed H_2_O_2_ cells for 4 h relative to the control as described in the materials and methods section.

**Figure 3.**
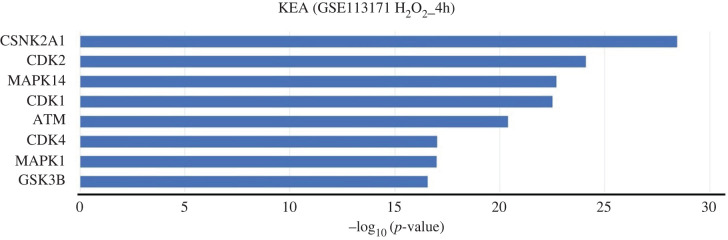
DNA damage and P38 signalling markers in H_2_O_2_-exposed cells. (*a*) Histogram of KEA results showing the main protein kinases driving the expression of DEGs following H_2_O_2_ treatment for 4 h (GEO accession: GSE113171).

The enrichment of protein kinases related to p38 signalling and DNA damage response was then investigated using KEA analysis in H_2_O_2_-treated cells. Similar to Bafilomycin A1-treatment, H_2_O_2_ treatment showed that MAPK14 and ATM are among the major enriched protein kinases driving expression of DEGs (figure 3). Consistently, oxidative stress has been shown to be one of the activators of the P38 signalling pathway, for instance, oxidative stress-mediated p38 signalling has been observed in HSCs (haematopoietic stem cells) [18] and neural stem cells [19] as a result of the loss of ATM protein kinase. Meanwhile, oxidative stress could induce oxidative DNA damage through increased ROS production [20].

### 2.4. Oxidative stress role in mediating the effect of autophagy suppression on elevation of p38 signalling and DNA damage response

I then hypothesized that the observed enrichment of p38 signalling and DNA damage response in autophagy-suppressed cells may be due to oxidative stress induction.

**Figure 4.**
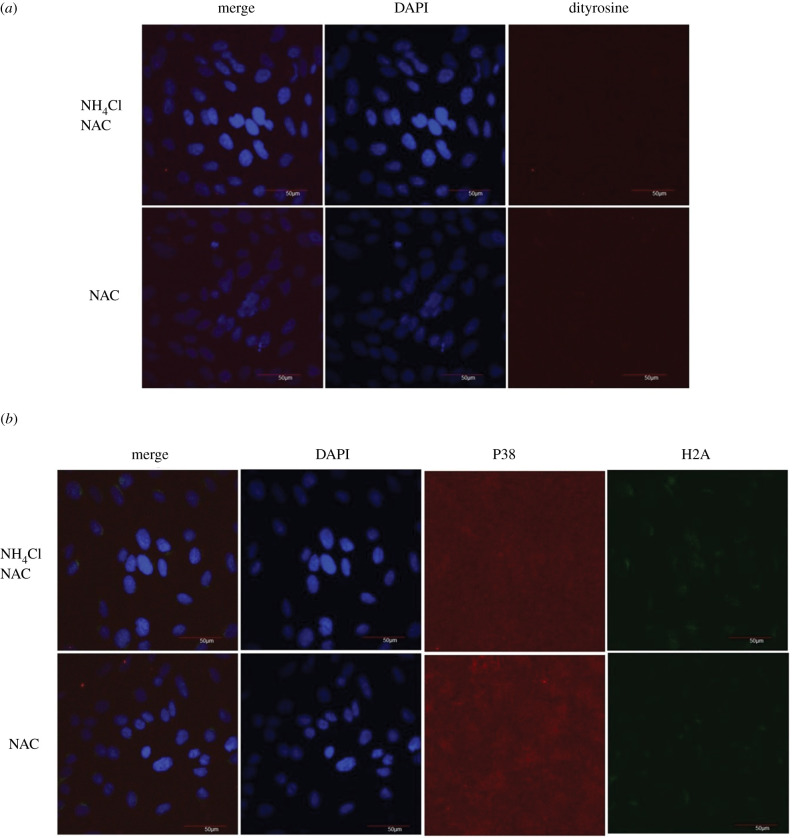
Oxidative stress inhibitor (NAC) treatment restores p38 signalling and DNA damage markers in fibroblasts. (*a*) Representative dityrosine immunostaining in NH_4_Cl-treated and/or NAC-treated groups (*b*) Representative phospho-H2A.X and P-p38 immunostaining in NH_4_Cl-treated and/or NAC-treated groups.

To order to investigate this point in detail, I measured dityrosine, phospho-H2A.X protein (DNA damage marker) and p38 signalling levels in Nh4cl exposed fibroblast cells with oxidative stress inhibitor (*N*-acetyl-l-cysteine (NAC)) treatment. Interestingly, the addition of an oxidative stress inhibitor (NAC) restored dityrosine staining compared to the control group. Furthermore, NAC treatment restored and alleviated p38 signalling and DNA damage marker (H2A.X) in NH_4_Cl-treated cells (figure 4). These results are consistent with previous studies showing that autophagy inhibition induces oxidative stress [15].

## 3. Discussion

Autophagy is a cellular catabolic process for lysosomal elimination of damaged cellular organelles and proteins. Autophagy inhibition leads to the disruption of many cellular biological processes and the accumulation of damaged cytoplasmic components. It, therefore, plays an important role in maintaining intracellular protein homeostasis and cellular integrity throughout the body [1–3].

Accumulation of damaged proteins may be responsible for the harmful effects of autophagy suppression. Here, I investigated other possible factors that mediate the adverse effects of autophagy suppression.

The crosstalk between autophagy and oxidative stress has shown that ROS are among the stress factors that activate autophagy, which in turn reduces ROS production through mitophagy [9,10,12].

Importantly, on the other hand, autophagy suppression caused oxidative stress, as we have seen here and in previous publications [13–15].

In this study, I performed RNA seq data analysis of autophagy-suppressed cells and hydrogen peroxide exposed cells. Interestingly, the results showed enrichment of p38 signalling and DNA damage markers in both bafilomycin A1-treated and H_2_O_2_-treated cells.

To investigate the possibility of oxidative stress involvement in the enrichment of p38 signalling and DNA damage markers in autophagy-suppressed cells, p38 signalling and DNA damage markers in autophagy-suppressed cells were measured in the presence and absence of an antioxidant. The results showed that NH_4_Cl treatment progressively increased P-H2A.X and P-p38 antibody staining, which was then restored to the normal levels following NAC treatment. Taken together, these results suggested the possible role of oxidative stress in mediating p38 signalling and DNA damage markers following autophagy suppression.

Although autophagy suppression was performed through neutralizing lysosomal pH (NH_4_Cl and bafilomycin A1) in this study, autophagy inhibition with 3-methyladenine (3-MA) in zebrafish larvae increased P-H2A.X and P-p38 antibody staining as well (A.E. 2020, unpublished data).

## 4. Methods

### 4.1. Data

The processed RNA seq data available on the GEO accession number GSE60570 were used to retrieve RNA seq count data of RPE-1 cells treated with bafilomycin A1 for 6 h relative to the control cells.

Fold change gene expression and DEGs (differentially expressed genes) were detected using the edgeR Bioconductor package (adj *p*-value < 0.01).

The raw RNA seq data available on the NCBI SRA accession number SRP140470 were used to retrieve DEGs between H_2_O_2_-exposed Hela cells for 4 h relative to the control.

Firstly, the SRR accession list (SRR7008199, SRR7008205, SRR7008211, SRR7008201, SRR7008202, SRR7008207, SRR7008208, SRR7008213, SRR7008214) was imported into galaxy Australia servers, and then fastqc tool was used to assess the quality of fastq files. The Per bas sequence quality graph of fastqc results showed that the end of reads has lower quality scores, therefore, the cutadapt tool was used to trim short reads (less than 20 bp) and poor quality bases (quality cutoff less than 20 bp).

Trimmed raw reads were then mapped to the human genome reference (Fasta file) obtained from the Ensemble database (http:/www.ensembl.org) using the HISAT2 tool on galaxy servers.

The RNA seq count table was created using the ‘Htseq-count’ and ‘generate count matrix’ tools on the galaxy servers using the GTF annotation file obtained from the Ensembl database.

DEGs (differentially expressed genes) were detected using the edgeR Bioconductor package (adj *p*-value < 0.01).

### 4.2. Data analysis

#### 4.2.1. Kinase enrichment analysis

The KEA analysis [21] was used to identify the main protein kinases driving the expression of DEGs in bafilomycin A1-treated RPE-1 cells and H_2_O_2_-treated cells.

#### 4.2.2. Gene set enrichment analysis

First, a pre-ranked gene list was produced using the edgeR package from the RNA seq count data. GSEA [22] was then used to test for enrichment against DNA damage response (MSigDB: M5464), MAPK pathway P38 (MSigDB: M12012) and Chuang oxidative stress response (MSigDB: M10970).

The gene sets were considered significant by *p*-value < 0.05 and *q*-value < 0.25.

#### 4.2.3. Enriched pathway analysis

I used the WikiPathways database [23] on ENRICHR to identify enriched signalling pathways. Pathways were considered significant at adj *p*-value < 0.01. Figure 1*c* displays the 10 main enriched pathways.

### 4.3. Cell line and culture maintenance

Zebrafish fibroblast cells (ATCC CRL-2298) were grown in DMEM/F12 mixture (Sigma-Aldrich, SLM-243-B) containing 10% fetal calf serum, 1% penicillin-streptomycin (Sigma-Aldrich, P4333) at 28°C and 5% CO_2_.

### 4.4. Drug treatment

NH4Cl (Sigma-Aldrich, A9434) was dissolved in ddH_2_O as a stock of 1.43 M and used overnight at a final concentration of 5 mM. NAC antioxidant (Sigma-Aldrich, A9165) was used overnight at a final concentration of 25 mM.

### 4.5. Fibroblast immunocytochemistry

Fibroblast cells (6 × 10^4^) were plated onto a 4-well chamber slide (Thermo Fisher Scientific, 154917-PK) 10 h before the treatment. NAC and/or NH_4_Cl have been incubated overnight. Cells were fixed at room temperature in PBS/4% PFA for 10 min. Slides were permeabilized at room temperature in PBS/0.2% Triton X-100 for 2 min and blocked in PBS/1% BSA for 1 h. Slides were then incubated in PBS/1% BSA with mice Dityrosine (SKU, SMC-520), mice phospho-p38 MAPK (Cell Signalling, 9216), and rabbit phospho-H2A.X (S139) antibody (Abcam, ab81299) overnight at 4°C. Slides were then washed three times in PBS and incubated in PBS/1% BSA with goat anti-rabbit secondary antibody, 488 (Thermo Fisher Scientific, A-11034), goat anti-mouse, 488 (Abcam, ab150113) or goat anti-mouse, 594 (Thermo Fisher Scientific, no. R37121) at a 1/200 dilution in the dark at room temperature for 2 h. For counterstaining, the cells were incubated with DAPI at a dilution of 1/250 in the dark for 30 min, and the slides were finally mounted using Fluoromount (Sigma, F4680).

## Data accessibility

This article has no additional data.
